# Extension of the Clinical and Genetic Spectrum in SPG4 Discloses a New Form of Alzheimer's Disease

**DOI:** 10.1002/alz70855_102713

**Published:** 2025-12-29

**Authors:** Emanuele Panza, Arun Meyyazhagan, Eliseo Picchi, Gustavo Ribas, Ingrid Faber, Ryosuke Miyamoto, Preethi Basavaraju, Haripriya Kuchi Bhotla, Mario Stasi, Francesco Patti, Filippo Maria Santorelli, Marcondes Cavalcante França, José Luiz Pedroso, Orlando Graziani Povoas Barsottini, Hélio Afonso Ghizoni Teive, Peter Henry St George‐Hyslop, Toshitaka Kawarai, Antonio Orlacchio

**Affiliations:** ^1^ University of Bologna, Bologna, Italy; ^2^ IRCCS University Hospital of Bologna, Bologna, Italy; ^3^ University of Perugia, Perugia, Italy; ^4^ University of Rome “Tor Vergata”, Rome, Italy; ^5^ Federal University of Paraná (UFPR), Curitiba, PR, Brazil; ^6^ University of Brasília, Brasília, Federal District, Brazil; ^7^ Tokushima University Graduate School of Biomedical Sciences, Tokushima, Japan; ^8^ Bharathiar University, Coimbatore, India; ^9^ University of Catania, Catania, Italy; ^10^ IRCCS Stella Maris Foundation, Calambrone, Pisa, Italy; ^11^ University of Campinas (UNICAMP), Campinas, SP, Brazil; ^12^ Federal University of São Paulo (UNIFESP), São Paulo, SP, Brazil; ^13^ Columbia University Irving Medical Center, New York, NY, USA; ^14^ Hyogo Prefectural Harima‐Himeji General Medical Center, Himeji, Japan; ^15^ IRCCS Santa Lucia Foundation, Rome, Italy

## Abstract

**Background:**

Hereditary spastic paraplegia (HSP) is a group of disorders characterized by progressive spasticity and lower limb weakness, with mutations in SPG4/*SPAST* being the most common cause. Detailed studies and clinical and molecular comparison across different populations are missing. Here we assessed the comprehensive spectrum of neurological features and screened for mutations in SPG4/*SPAST* gene in patients with HSP.

**Method:**

SPG4/*SPAST* variants were detected using Direct and Next‐Generation Sequencing. The pathogenicity of novel variants was confirmed through familial segregation and *in silico* analysis. The study involved 726 HSP patients (628 familial, 98 sporadic) recruited from Italy, Brazil, and Japan between 2001 and 2024, with analysis conducted in collaborative centers.

**Result:**

Our study highlights clinical and epidemiological differences between populations, particularly in phenotype, age of onset and disability, expanding the SPG4 clinical spectrum. Genetic analysis found 52 pathogenic SPG4/*SPAST* mutations in 284 patients, including 20 missense mutations, four of which were novel. Many mutations were population‐specific, with a possible founder effect for a recurrent variant in Italy. Notably, 28 HSP‐SPG4 patients exhibited a novel form of Alzheimer's disease (AD), confirmed by autopsy in four cases. Additionally, 18 patients with SPG4/*SPAST* mutations had a thin *corpus callosum* and intellectual disability.

**Conclusion:**

In this study, we investigated pathogenic SPG4/*SPAST* variants in an international cohort of HSP patients from three continents. Our results extend the clinical spectrum of HSP‐SPG4, discovering a new type complicated by an atypical form of AD. Cautious evaluation of genotype‐phenotype relationships proposes insights into patient counseling and future research planning.

